# Implementing a Personalized Nutrition Service in a Real-Life Military Setting: Feasibility and Consumer Acceptance

**DOI:** 10.1016/j.cdnut.2026.109533

**Published:** 2026-09-18

**Authors:** Sandra van der Haar, Meeke Ummels, Suzan Wopereis, Femke P M Hoevenaars, Imre W K Kouw, Martijn W J Noort

**Affiliations:** 1 Wageningen Food & Biobased Research, Wageningen University & Research, Wageningen, The Netherlands; 2 Microbiology and Systems Biology Department, Netherlands Organization for Applied Scientific Research (TNO), Leiden, The Netherlands; 3 Division of Human Nutrition and Health, Nutritional Biology, Wageningen University & Research, Wageningen, The Netherlands; 4 Department of Intensive Care Medicine & Research, Gelderse Vallei Hospital, Ede, The Netherlands

**Keywords:** personalized nutrition, feasibility, implementation, 3D food printing, prototype evaluation, dietary advice system, user experiences, product evaluations

## Abstract

**Background:**

Despite growing evidence that personalized nutrition (PN) can improve dietary behavior and health outcomes, the implementation of PN food services in real-world settings remains largely unexplored.

**Objectives:**

This study evaluated the feasibility of a PN service to deliver personalized nutrition products (PNPs) in a military setting and assessed consumer acceptance following repeated consumption.

**Methods:**

A PN service was developed to design, produce, and distribute PNPs. Twenty-five participants from the Royal Netherlands Army took part in a feasibility study, using a single-cohort pretest posttest design. Each participant consumed a PNP (in-between meal) on 10 consecutive days during a combat training exercise. Product liking was assessed through daily questionnaires, whereas broader user experiences were assessed at baseline (*t* = 0), 1-wk follow-up (*t* = 1), and 2-wk endline (*t* = 2).

**Results:**

The PN service was successfully implemented during a military training exercise. In total, 229 PNPs were produced and distributed. Participants evaluated all aspects of the PN service as slightly positive (4.5 ± 1.4 to 5.0 ± 1.7 on 7-point scales), except for variation in products, which was rated as slightly negative (3.4 ± 1.6). Across the different product combinations, mean overall liking and all sensory attributes were slightly positive (5.7 ± 1.7 to 6.1 ± 1.6 on 9-point scales). Overall product liking did not change over time (*F* (9, 194) = 1.242, *P =* 0.27). Attitudes toward a PNP service in the army were positive at study start but decreased to neutral at study end.

**Conclusions:**

This study supports the feasibility of a prototype PN service in a real-life military setting. The PNPs received modestly positive evaluations, and repeated exposure did not change the overall liking of the products. Initial positive attitudes toward the service declined over time. Therefore, further optimization of both the service and the PNPs is needed before implementation at a larger scale can be considered.

## Introduction

Personalized nutrition (PN) is founded on evidence-based science and uses individual-specific data to improve dietary behaviors and health outcomes [1]. Several studies have shown that PN interventions are more effective than generic interventions using a “one-size-fits-all” approach in improving dietary habits [[2], [3], [4], [5]] and health outcomes such as cardiometabolic profile [[6], [7], [8]], postprandial blood glucose levels [9], and gastrointestinal (GI) complaints [10]. In addition, personalized and precision nutrition are emerging fields in sports and military nutrition by tailoring nutritional strategies to individual characteristics, performance goals, and training or operational demands, although intervention evidence remains limited [[11], [12], [13]]. A key challenge in translating PN into practice is that most existing services are limited to provide dietary advice, meal plans, or grocery recommendations [14]. Emerging evidence suggests that embedding personalized recommendations directly in foods and food services may facilitate adherence and potentially enhance health impact compared with advice-only approaches [15]. However, the translation of individual health-related data into tailored food products that can be produced and delivered at scale remains largely unexplored, and few studies have investigated how personalized nutrition products (PNPs) can be developed, delivered, and consumed within real-world environments. Within the IMAGINE research consortium (website IMAGINE—Health-Holland) [16], this gap has been addressed by the development of a prototype PN service that translates individual nutritional requirements into personalized in-between meals. This PN service integrates the generation of personalized dietary advice with the design, production, and distribution of customized in-between meals, thereby providing a unique opportunity to evaluate how PN can be implemented in practice [17]. An important element of the service is 3-dimensional (3D) food printing. As an additive manufacturing process, 3D food printing enables foods to be produced layer-by-layer according to a digital design, allowing nutrient composition, portion size, shape, and functionality to be tailored to individual requirements [14,18,19].

The military represents a particularly relevant setting in which to evaluate such an approach, as personnel often operate under physically and mentally demanding conditions that result in substantial variability in nutritional requirements [20]. Tailored nutrient compositions and functional ingredients may offer specific benefits for these specialized requirements. For example, physical and mental health issues are common in military personnel, such as gastrointestinal problems [21], musculoskeletal injuries [22], posttraumatic stress disorder, sleep problems, and depression [23,24]. Providing optimal nutrition to soldiers through PN might therefore contribute to physical and mental health, including aspects such as improving vigilance and physiological recovery [25], which may ultimately improve overall performance.

Beyond the technical feasibility, successful implementation of PN services depends largely on consumers’ acceptance and sustained use. Previous research has shown that innovations in food have high failure rates (∼50%) [26], partly due to unforeseen consumer reactions [27]. To be accepted, a new technology or novel food must demonstrate value in terms of nutrition and good health it may offer [28]. For PN specifically, acceptance is likely influenced by the perceived relevance and value of the tailored product to the individual user [29]. Caulier and colleagues were the first to investigate the perception of actual 3D-printed food products in a military setting. Their findings demonstrated that repeated consumption of a 3D-printed cereal-based recovery bar increased participant satisfaction and acceptance and suggested that the perceived degree of personalization contributed to these outcomes [30]. However, user evaluations of an integrated PN service encompassing product development, production, delivery, and consumption have not yet been performed.

Therefore, this study aimed to assess the feasibility of implementing a 3D food printing-based PN service in a real-life military environment. As consumer acceptance is an important determinant of successful implementation, product liking, consumer intentions, and attitudes toward PNPs after repeated consumption were assessed.

## Methods

### Participants

Participants were recruited among different military units of the Royal Netherlands Army. In coordination with the army commanders, an infantry training unit of 40 soldiers located in and around a military base in Oirschot, the Netherlands was selected based on the criterion of being based at the same place for a duration of 2 wk. Soldiers within this unit were approached by the researchers to participate in a field laboratory. An information meeting was organized before the start of the study, in which participants were informed, provided written informed consent, and were checked for eligibility. Inclusion criteria were: Dutch speaking and ≥16 y of age. Exclusion criteria included having food allergies or intolerances or other medical contraindications as decided by a military physician.

A cohort of 25 soldiers enrolled for participation in a 2-wk study period, during which they were taking part in infantry training. The training program prepared them for placement at a combat unit and consisted of offensive and defensive combat training. The first part of the training program (in week 1 of the study) took place at the military base of Oirschot, Harskamp, and Stroe, with activities including practice of shooting at the shooting range, performing sports, and realistic combat training. The second part of the training program involved field training that took place in the woods close to the military base of Oirschot, where the soldiers were sleeping outside for 3 consecutive nights, with a maximum of 4 h of sleep per night.

### Study design

The study followed a single-cohort pretest posttest design, with a duration of 2 wk, and was executed between 27 May and 7 June, 2024. In this proof-of-concept study, the PN service developed within the IMAGINE research consortium was evaluated in terms of consumer experience and product liking in a real-life setting. In addition to a military setting, the service was also tested in a medical setting [31]. The PN service contained several elements: *1*) *a digital application* for consumers to provide personal data, indicate taste preferences, and to receive the health feedback on which the PNPs were based; *2*) *a dietary advice algorithm*, in which the aforementioned personal data were used as input to generate dietary advice based on evidence-based knowledge rules; and *3*) *a prototype Flexible Food Manufacturing Platform (FFMP)—*which is a mini food factory using 3D food printing and dosing stations for various food materials and supplement powders that automatically produced the PNP based on the dietary advice algorithm. The service produced fresh personalized in-between meals (for participants referred to as: “Nutri-Bites”) that were tailored to an individual’s needs and preferences, containing nutrients that support evidence-based health benefits. Noort et al. [17] describe in detail how the PN advice system was designed and developed, including the knowledge base for the underlying personalized dietary advice, development of food materials for the PNPs, and engineering of the prototype FFMP. The intervention consisted of daily PNP consumption and product evaluation using questionnaires for 10 consecutive working days (Figure 1). Before each working day, the digital application collected personal participant data as input for the next day’s PNP. Weekend days were not included in the evaluation. As this was a feasibility study and the aim was to test the prototype and evaluate user experiences in a real-life setting, no control group was included.

**FIGURE 1:**
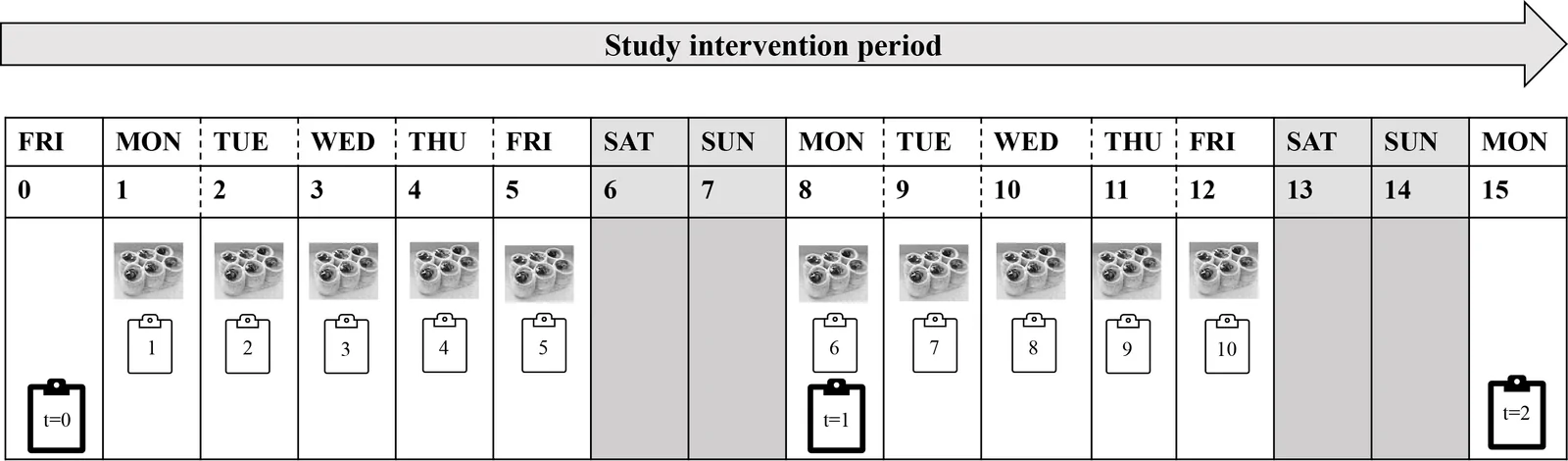
Overview of study design. In this 10-d study, a PNP was distributed and assessed on weekdays in *n* = 25 soldiers by using daily questionnaires after product consumption. At baseline (*t* = 0), after 5 d of PNP consumption (*t* = 1) and at the end of the study period (*t* = 2), a survey was administered to assess self-perceived consumer attitudes, intentions, perceptions toward personalized foods, health, and well-being. PNPs, personalized nutrition products.

### Study procedures

During the study period, a FFMP was located at the Netherlands Organisation for Applied Scientific Research (TNO) premises at the High Tech Campus in Eindhoven, the Netherlands, close to the military base. The FFMP produced daily PNPs on the basis of personalized dietary advice, summarized in a printer code. The advice underlying the PNP was generated with several fixed and dynamic input parameters. Body weight, height, and wrist circumference were measured (for determining body type: ectomorph, mesomorph, or endomorph) and sex, age, average daily physical activity level (PAL), vitamin D level, and habitual fiber intake were collected through a survey during a screening visit before study commencement as fixed parameters per subject. Dynamic parameters, such as specific daily PAL data, GI complaints, coffee and energy drink intake, sleep quality, and taste preference, were collected daily through a digital application (TNO Diamonds dashboard) that was accessible for participants on study tablets. Daily PAL data were filled out in a physical activity planner by the group commander for each individual soldier and then transferred to the application. All the aforementioned parameters were used as input for the PNP on the following day. Total daily PAL was determined by combining general PAL with the individual daily PAL values for specific training activities over a 24-h period, as recorded in a physical activity log provided by the group commander.

Each day, after all input parameters were collected, 25 printer codes were generated and sent to the FFMP. The PNPs were produced on the next morning between 08:00 and 13:00. After production, the PNPs were transported to the military base in a cooled transportation box. The PNPs were distributed in disposable containers as an in-between meal product between lunch and dinner, and consumption always took place in the afternoon between 14:00 and 16:00. Participants were informed about the research aims but not on the exact production process of the PNPs that included the 3D-printer (incomplete disclosure) to minimize risk of bias in the sensory evaluations. During product consumption, participants filled out a short questionnaire evaluating their PNP on several sensory aspects such as overall liking, taste, and texture. Furthermore, participants indicated how much of the product they had eaten (they were not obliged to consume the whole product) and reported GI complaints if any had followed after consumption of the PNP the day before. Besides these daily questionnaires, participants were asked to fill out a more detailed questionnaire at baseline (*t* = 0), after 5 d of PNP consumption (*t* = 1), and at the end of the study (*t* = 2), assessing their attitudes, intentions, perceived benefits, and barriers toward a personalized food service in the army.

### Personalized nutrition products

#### Health benefits and nutritional advice

Baseline data profile, daily questionnaires, and daily PAL were integrated into the selection of a “health benefit” which served as input for the PNP of the next day. The predefined health benefits comprised *1*) immune health, *2*) digestive health, *3*) endurance, *4*) muscle power, *5*) recovery, and *6*) mental performance. The daily health benefit selection protocol worked as follows: on the first day, participants were assigned based on either a high score (4–5 points) on the immunity questionnaire or a low fiber intake (<22 g/d according to the fiber screening questionnaire). If neither of these conditions applied, the health benefit that best matched the main activity planned for the day was chosen. For all consecutive days, assignment of the same health benefit was restricted to a maximum of 2 consecutive days; “recovery” was mandatorily assigned on a day succeeding “muscle power,” and any extreme outcomes from daily questionnaires superseded activity-based selection criteria. Nutritional recommendations associated with each health benefit are described in Supplemental Table 1A. Micronutrients were selected based on a literature review, taking the principles of the evidence pyramid as a guideline [32]. Nutrients were selected in case a health claim, meta-analysis, or convincing evidence from randomized controlled trials was present. Macronutrient composition and sizing of the product were based on the level of activity in case of muscle power, endurance, or recovery. In cases where digestion, immunity, or mental performance were assigned as health benefits, body type determined macronutrient composition and product size. A detailed description of the personalized dietary advice, including the scientific rationale for nutrient and ingredient selection, was beyond the scope of the present manuscript and is reported elsewhere [17]. Participants received daily feedback in the online portal regarding the health benefit that was assigned for them for the next day. The information was presented to them as follows: “Tomorrow, you will receive a Nutri-Bite that supports your immune system, as you mentioned you are feeling unwell *(example)*.”

#### Product design

Each PNP consisted of *1*) *a casing*: an edible multicompartment container with fillable cavities, made out of a variety of doughs or pastes; *2*) *a filling*, which was fruit-, yogurt-, custard-, cheese-, peanut-, or chocolate-based; and *3*) *a topping,* containing a flavor that was selected by the participant (chocolate, caramel, summer fruit, strawberry, green apple, orange, or lemon). In addition, micronutrient powders were dosed in the bottom of the casing cavities of the PNP to fulfill a specific micronutrient composition. Figure 2 shows a visual example of a PNP as presented to the participants. The product composition of each PNP (casing, filling, topping) was based on the macronutrient and micronutrient composition for each health profile as described in Supplemental Table 1B, which was in turn determined by the fixed and flexible input parameters specifically related to the individual participant and their specific activity and conditions. Some input parameters (such as body type, PAL data, and dosing of a certain ingredient) also affected the size of the PNP (6 or 8 cavities).

**FIGURE 2:**
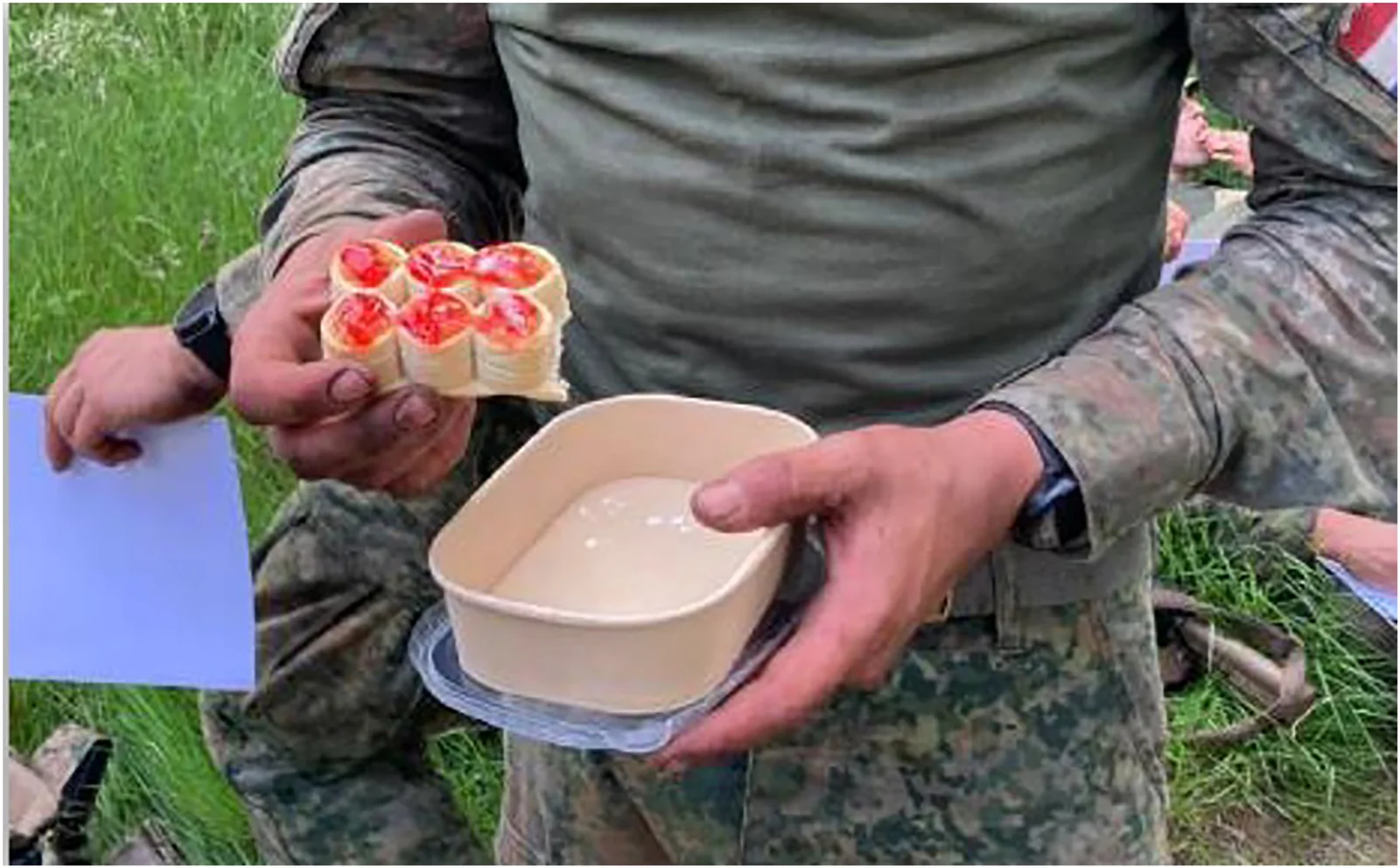
Photo of a PNP as presented to the participants, consisting of a baked dough casing, filling with incorporated micronutrient powder, and topping to match a specific macronutrient and micronutrient composition per health profile. PNPs*,* personalized nutrition products.

#### Production process

PNPs were produced using the FFMP, a fully automated mini food factory. Dough casings were 3D printed and baked at 150°C for 2, 4, or 12 min, according to dough type, then cooled to a core temperature below 40°C. Micronutrient powders were dosed to the casing cavities, followed by the appropriate filling and participant-selected topping. Finished PNPs were packed in a disposable carton box, labeled with a participant identifier and stored at 5°C before transport in cooled boxes to the military base. All products were consumed within 8 h after production to ensure a fresh product texture and microbial safe product.

### Study measures

#### Product consumption and sensory evaluation

Several sensory parameters of the PNPs were assessed daily using a questionnaire. Participants rated the PNPs on appearance, mouthfeel, taste, aftertaste, freshness, and overall liking on a 9-point Hedonic scale ranging from “dislike extremely (1)” to “like extremely (9).” After rating these attributes, participants filled out an open question (optional) on how, in their opinion, the PNP could be improved. Hereafter, participants were asked about product consumption (*I have consumed the product completely, “yes” or “no”*). In case participants did not consume the entire product, they were asked to specify the reason, selecting from the following options: not hungry, did not like the product, or other. Finally, participants were asked to indicate how likely it was that they would be willing to consume this specific PNP again, on a 7-point Likert Scale ranging from “very unlikely (1)” to “very likely (7)” and to fill out a reason (optional).

#### GI complaints

GI complaints after PNP consumption were assessed daily, except on the first study day as participants had not yet consumed a PNP. Participants were asked the following question: “*After consumption of the PNP yesterday, did you experience any gastro-intestinal complaints such as bloating, belching, nausea, flatulence, diarrhoea or constipation?*” If they indicated yes, they were asked to describe which type of complaint they experienced.

#### Attitudes, intentions, and perceptions toward personalized food service

At 3 time points, *t* = 0, *t* = 1, and *t* = 2, participants completed questionnaires assessing their attitudes, intentions, and perceptions toward a personalized food service. All items were rated on a 7-point Likert Scale. In the baseline questionnaire, the concept of the personalized food service was first explained to them as they had not yet seen it. Participants then completed items measuring their general attitude and perceived value toward a personalized food service in the army (1 = not at all valuable, 7 = very valuable), as well as their intention to use. Intention items reflected the 3 stages of behavior change: precontemplation, contemplation, and action [33], assessed by using the following phrase: *I would consider to make use of it, I plan to make use of it, I certainly would make use of it* (1 = completely disagree, 7 = completely agree).

At both *t* = 1 and *t* = 2 questionnaires, the same attitude and intention items were repeated to assess changes over time. In addition, participants rated their expectations regarding potential health benefits on PNP consumption (e.g., on digestion, alertness, immune system, recovery, endurance, and muscle strength). Finally, they also evaluated potential benefits and barriers of PNPs, including aspects such as taste, freshness, feasibility of implementation within the army setting, time investment, and use of personal data.

#### Self-rated health and well-being

At *t* = 0, *t* = 1, and *t* = 2, participants were asked to rate their physical and mental health. Self-perceived overall health was measured with the Self-Rated Health scale, a widely used single-item measure developed by Ware and Sherbourne (1992), that encompassed the following question: “*How would you currently rate your own health on a scale from 0 to 100, where 0 is very poor health and 100 is excellent health*?” Mental health (well-being) was measured with the WHO-5 Well-being Index. This index consists of 5 statements on well-being referring to the past 2 wk. Each statement is rated on a 6-point scale, ranging from “at no time (0)” to “all of the time” (5) with higher scores indicating better well-being. The scores on each statement make up a subscore, which is multiplied by 4 to give a total score, with 0 representing the worst imaginable well-being and 100 representing the best imaginable well-being. Total scores below 50 indicate poor well-being.

#### Demographics and other study sample characteristics

From the baseline questionnaire that was used as input for the PNP, sociodemographic characteristics of the participants were collected, such as sex, age, BMI, and habitual fiber intake. Furthermore, habitual in-between meal behavior (frequency and type of food products) and food choice motives of all participants were included from the *t* = 0 questionnaire. Consumer choice was assessed from a predefined list of 12 motives (healthy, animal friendly, safe, natural, easy to use, affordable, ethical concern, sensory appealing, familiar, mood, environment, and weight management) [34]. Participants were asked to rank each of these food choice motives on 7-point Likert scales, ranging from “very unimportant (1)” to “very important (7).”

Habitual in-between meal consumption was assessed with questions on the frequency of eating in-between meals (“yes” or “no”) and if yes, to indicate how many in-between meals they consumed on average per day.

### Statistical analyses

Data on PNP composition and sensory ratings were presented as means ± SD. Categorical variables (GI complaints, product consumption) were displayed as frequencies and percentages. Responses to the open-ended question regarding potential improvements to the PNP were categorized into overlapping topics and summarized by calculating the frequency of each category. A general linear model (GLM) with time as a fixed factor and participant as a random factor was conducted to assess the effect of repeated consumption on overall liking scores of all PNPs. Because this was a feasibility study with limited numbers of observations for each combination of product characteristics, the assumptions to test the effect of repeated consumption of specific filling, casing, and topping combinations in a linear mixed model were not met. To test the difference in mean Likert scores over time (*t* = 0, *t* = 1, *t* = 2) for attitudes, intentions, self-rated health, and well-being, repeated-measures ANOVA was applied. Subsequently, least significant difference post hoc tests were performed to assess the differences between timepoints. To test the difference in mean Likert scores for benefits and barriers of PNPs between *t* = 0 and *t* = 2, paired samples *t*-tests were applied.

Sensory ratings of dropouts were included in the sensory analyses and in the GLM. In the overtime analyses of the *t* = 0, *t* = 1, and *t* = 2 questionnaires, dropouts were excluded. Statistical significance was set at *P <* 0.05 for all analyses. All statistical analyses were performed using SPSS software (version 25.0; IBM Corp).

## Results

### Participants and baseline characteristics

A total of 25 male participants were included in the study with a mean age of 20.7 ± 2.0 y (18–25) and BMI 24.1 ± 1.9 kg/m^2^ (21.2–27.6). Habitual fiber intake at baseline averaged 14.8 ± 7 g/d, which is below the Dutch recommendations for males, that is, 40 g/d. The majority of participants (92%) regularly ate in-between meals in their daily diet with a mean of 2.7 ± 1.3 in-between meals per day. On the basis of the predefined list of 12 Food Choice Motives, the top 5 most important motives for food choice were: sensory appealing (6.0 ± 1.1), “gives me a good feeling (mood)” (5.7 ± 1.1), affordable (5.5 ± 1.4), safe (5.3 ± 1.7), and healthy (5.3 ± 1.0). Five participants (20%) dropped out during the 2-week study period, which coincided with the most intensive sleep deprivation phase of the military training. All dropouts reported not liking the study products as the main reason for quitting the study, in combination with experiencing GI complaints (*n* = 2/5) or having expectations of personal health benefits that were not met (*n* = 1/5).

### Implementation of the PN service

#### Product consumption

The PN service was successfully implemented during a military combat training exercise of 2 wk, and a total number of 229 PNPs were produced and distributed to participants during the study on 10 subsequent working days. Of these, 208 PNPs (91%) were consumed entirely, and 21 PNPs (8%) were consumed partly. GI complaints were reported 24 h postconsumption. Complaints were reported by 9 of 25 participants (36%) and in 14% of the daily questionnaires (*n* = 33/229). Flatulence or bloating (41%), stomach pain (19%), and nausea (19%) were the most reported GI complaints.

#### Evaluation of the PN service

At the end of the 2-wk period, the following aspects were scored as slightly positive: the number of choices in topping flavors (4.5 ± 1.4), the time between ordering and distribution of the PNP (4.6 ± 1.2), the amount of personal information that had to be shared to produce the PNP (4.8 ± 1.5), the amount of time spent on ordering a PNP (5.0 ± 1.7) and the user-friendliness of the application for ordering (5.0 ± 1.7). The only aspect of the PN service that scored slightly negative was the variation in PNPs (3.4 ± 1.6).

### Product evaluations

Table 1 shows the sensory evaluation of all distributed PNPs without making a distinction in product characteristics. The mean scores for overall liking, appearance, mouthfeel, taste, aftertaste, and freshness were all slightly positive (ranging from 5.7 ± 1.7 to 6.1 ± 1.6). Willingness to consume the specific PNP again received a neutral score (4.2 ± 1.7).

**TABLE 1:** Sensory evaluation on taste and texture attributes, and overall liking for all PNPs (*n* = 229)

Sensory aspect	Mean score (SD)^1^
Overall liking	5.9 (1.6)
Appearance	6.0 (1.3)
Aftertaste	5.7 (1.7)
Freshness	6.0 (1.4)
Mouthfeel	5.8 (1.6)
Taste	6.1 (1.6)
Willingness to eat again	4.2 (1.7)^2^

Abbreviation: PNP, personalized nutrition product.

1 Assessed on a 9-point Hedonic scale, ranging from extremely unappealing (1) to extremely appealing (9).

2 Assessed on a 7-point Likert Scale, ranging from not at all (1) to very much (7).

Table 2 displays the overall liking score per specific casing and filling combination. Overall liking scores of the PNPs for specific casing and filling combinations ranged from 2 to 9. The protein casing combined with custard filling received the highest rating (8.4 ± 0.5), whereas the plum paste combined with a peanut butter filling received the lowest rating (5.3 ± 1.4).

**TABLE 2:** Overall liking score for specific casing and filling combinations within PNPs

Casing type (dough/paste)	Filling type	Observations (*n*)	Mean score (SD)^1^
Protein	Custard	8	8.4 (0.5)
Protein	Peanut butter	51	5.7 (1.5)
Carrot	Ganache	24	5.5 (1.7)
Plum	Peanut butter	32	5.3 (1.4)
Plum	Custard	21	5.7 (1.6)
Cereal	Yogurt	15	5.6 (1.4)
Cereal almond	Apple	26	5.9 (1.8)
Cereal almond	Custard	4	7.0 (0.0)
Cereal high fiber	Peanut butter	26	6.0 (1.5)
Cereal high fiber	Banana-strawberry	15	7.2 (1.2)

Abbreviation: PNPs, personalized nutrition product.

1 Assessed on a 9-point Hedonic scale, ranging from extremely unappealing (1) to extremely appealing (9). The addition of powders containing specific micronutrient combinations was not taken into account.

Responses to the open-ended question on how the PNPs could be improved indicated that the most frequently mentioned areas for improvement were the filling (29%) and texture (27%). Comments related to the filling predominantly concerned the peanut butter formulation, which participants reported receiving too frequently or did not prefer. Texture-related comments referred mainly to the hardness, softness, stickiness, and moisture content of the products. Representative comments included: *“The texture of the dough was too soft and sticky; I would prefer a firmer texture,” “Too much peanut butter,”* and *“I do not like peanut butter!”*

#### Overall liking over time

The overall liking scores per day were reported within a range of 5.4 ± 1.5 (lowest mean score on day 1) and 6.5 ± 1.8 (highest mean score on day 9) on a 9-point Hedonic scale (Figure 3). However, no time effect on overall liking scores was detected (*F* (9, 194) = 1.242, *P* = 0.27).

**FIGURE 3:**
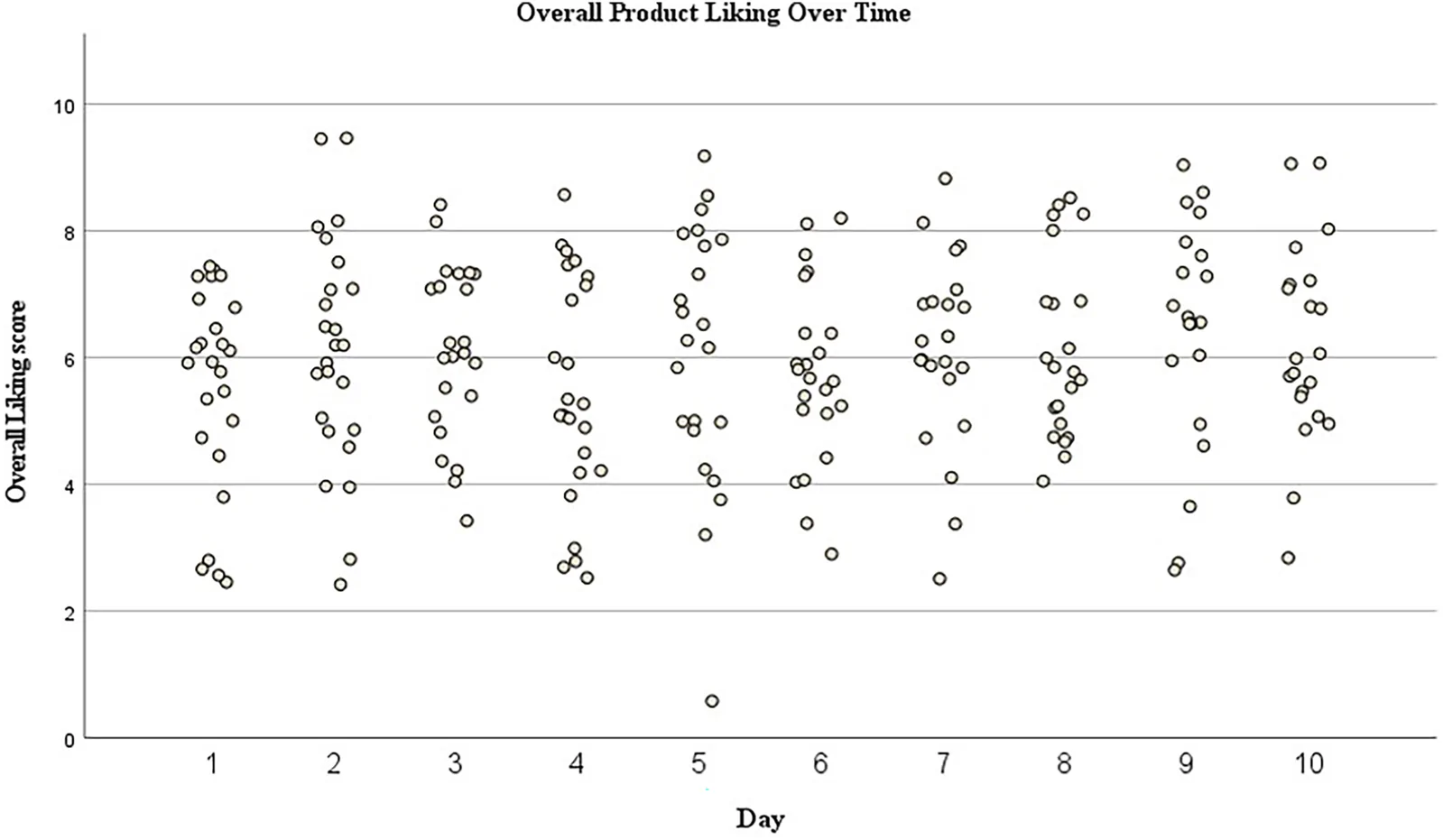
Overall liking scores for PNPs over the 10-d study period. PNPs, personalized nutrition products.

#### Attitudes, intentions, and perceptions during the intervention period

Table 3 shows the mean scores per time point for consumer attitudes, intentions, and perceived benefits and barriers. The mean score for attitude toward PNPs in the army significantly decreased over time (*F* (2, 38) = 11.5, *P* < 0.001), from a positive to a neutral score at the end of the intervention period. Intentions to use PNPs also decreased significantly over time, for the precontemplation stage (*F* (2, 34) = 13.2, *P* < 0.001), contemplation stage (*F* (2, 34) = 5.1, *P* = 0.01), and action stage (*F* (2, 34) = 8.8, *P* < 0.001) from positive toward slightly positive and neutral scores.

**TABLE 3:** Consumer attitudes, intentions to use, and perceived benefits and barriers, measured at baseline, after 1 wk, and after 2 wk

Survey item	Mean score (SD) on 7-point Likert Scales			
	*t* = 0 wk	*t* = 1wk	*t* = 2 wk	*P* value
Attitude toward PNPs				
“A personalized food service in the army is valuable”	5.9 (1.0)^a^	5.0 (1.4)^b^	4.5 (1.6)^b^	<0.001
Intention to use PNPs				
“I would consider it” (precontemplation stage)	5.9 (1.1)^a^	5.4 (1.1)^b^	4.3 (1.9)^c^	<0.001
“I am planning to” (contemplation stage)	5.7 (1.1)^a^	4.9 (1.2)^b^	4.6 (1.9)^c^	0.01
“I will definitely” (action stage)	5.8 (1.2)^a^	4.8 (1.4)^b^	4.0 (2.2)^c^	<0.001
Perceived benefits and barriers of PNPs				
PNPs are tasty	4.8 (1.2)		4.4 (1.3)	0.35
PNPs are fresh	5.3 (1.0)		4.7 (1.1)	0.06
PNPs are difficult to implement in the army	4.1 (1.5)		5.0 (1.5)	0.08
PNPs are time-consuming	3.4 (1.6)		4.4 (1.1)	0.02
I have concerns about sharing my personal data for PNPs	2.3 (1.4)		2.4 (1.3)	0.88

a,b,c – Different letters indicate differences between time points, *P* < 0.05.

Participants were on average slightly positive about the tastiness of the PNPs at baseline, and this did not differ after the 2-wk intervention period (*t =* 0.95*, P* = 0.35). The same holds true for the freshness of the PNPs, although the score trended lower after the intervention. At the start of the intervention, participants thought PNPs were not time-consuming; however, this increased over time to being significantly more neutral (*t =* –2.6*, P* = 0.02). Participants were neutral on whether it would be difficult to implement PNPs in the army at the start of the intervention, and at the end of the intervention they slightly agreed to this statement (*t* = –1.84*, P* = 0.08). Participants had no concerns on sharing their personal data to produce PNPs at the start of the study, and this did not change over time (*t =* –0.160*, P =* 0.88).

Table 4 shows the mean scores per time point for attitudes toward potential health benefits of PNPs, self-rated health, and well-being. Mean scores for attitudes toward potential health benefits of PNP reduced significantly over time for PNPs with health profile immune health (*F* (2, 36) = 3.7, *P* = 0.04), recovery (*F* (2, 36) = 11.9, *P <* 0.001), endurance (*F* (2, 36) = 7.0, *P* = 0.009), and muscle power (*F* (2, 36) = 15.5, *P <* 0.001). For all of these health profiles, participants scored (slightly) positive at baseline and more neutral toward the end of the study. For the health profile digestion, participants were slightly positive (5.0 ± 1.1), and this did not change over time (*P =* 0.27). For the health profile alertness, the mean score was also slightly positive at baseline (4.7 ± 1.5), and more neutral toward the end of the study (3.8 ± 1.5), with a nearly significant decrease over time (*P* = 0.06).

**TABLE 4:** Attitudes toward potential health benefits of PNPs, self-rated health and well-being, measured at baseline, 1 wk after the intervention, and 2 wk after the intervention

Survey item	Mean scores (SD)			
	*t* = 0 wk	*t* = 1 wk	*t* = 2 wk	*P* value
I think PNPs can have benefits for my…^1^				
Digestive health	5.0 (1.1)	4.5 (1.3)	4.8 (1.1)	0.27
Alertness	4.7 (1.5)	4.1 (1.6)	3.8 (1.5)	0.06
Immune health	5.1 (1.4)^a^	4.2 (0.9)^b^	4.2 (1.2)^b^	0.04
Recovery	5.7 (1.2)^a^	4.7 (1.1)^b^	4.2 (1.1)^b^	<0.001
Endurance	5.2 (1.5)^a^	3.9 (1.5)^b^	4.1 (1.4)^b^	0.009
Muscle power	5.3 (0.9)^a^	3.9 (1.5)^b^	4.1 (1.4)^b^	<0.001
Self-rated health score^2^	78 (12)	79 (11)	76 (11)	0.40
Well-being index score^3^	63 (11)^a^	65 (15)^a^	53 (10)^b^	0.002

a,b – Different letters indicate differences between time points, *P* < 0.05.

Abbreviation: PNP, personalized nutrition product.

1 Measured on 7-point Likert scales, ranging from strongly disagree (1) to strongly agree (7).

2 Measured on a scale ranging from 0 (worst possible health) to 100 (best possible health).

3 Index score ranging from 0 (worst possible quality of life) to 100 (best possible quality of life).

Self-rated health scores remained stable over time, with mean values of 78 ± 12 at baseline and 76 ± 11 after 2 wk of the intervention (*F* (2, 32) = 0.9, *P* = 0.40). In contrast, well-being index scores decreased throughout the intervention period, from 63 ± 11 to 53 ± 10 (*F* (2, 34) = 7.8, *P* = 0.002).

## Discussion

### Principal findings and discussion

This study demonstrated the feasibility of implementing a 3D food printing-based PN service in a real-life military setting. A prototype service encompassing personalized dietary advice, product design, production, distribution, and consumption was successfully operationalized within a population characterized by substantial variation in nutritional requirements. In total, 229 PNPs were delivered during a demanding military training period, providing proof of concept that individual nutritional recommendations can be translated into tailored food products and implemented under real-world conditions. As consumer acceptance is a critical determinant of successful implementation, users’ experiences with both the products and the service were evaluated. The acceptability of both the service and PNPs was modestly positive. Overall liking and sensory attribute ratings were slightly positive for all product combinations, indicating that participants generally accepted the products despite their novelty and the demanding context in which they were consumed. Importantly, none of the evaluated product combinations received mean negative ratings. Because of the personalized nature of the intervention, participants received products that differed in characteristics, making direct sensory comparisons challenging. Nevertheless, grouping products according to casing and filling combinations showed differences in liking between product types, suggesting opportunities for further optimization of the product formulations.

A limited number of studies have previously conducted sensory evaluations of 3D-printed food products. In a consumer study among military personnel, Caulier et al. [30] reported comparable overall liking scores for 3D-printed cereal-based recovery bars, ranging from 5.7 to 5.9 on 9-point Hedonic scales, depending on bar type. These scores are similar to our mean overall liking score of 5.9 for the PNPs in this study. Comparable findings have also been reported outside military settings. Talens and Rios [35] evaluated 3D-printed breakfast bars in a panel of Spanish older adults. Although concept and appearance were initially rated positively (6.0 ± 2.0 on a 9-point scale), acceptability decreased significantly to a neutral score after tasting (5.0 ± 2.0), suggesting that the sensory experience did not meet expectations of the product concept. Khemacheevakul et al. [36] evaluated the sensory profile of several sugar-reduced and nonsugar-reduced 3D-printed chocolates. Liking scores ranged from 6.4 ± 1.6 to 6.9 ± 1.5. Their results showed that layering high- and low-sugar chocolate via 3D printing enabled ≤19% sugar reduction without adversely affecting perceived sweetness or overall liking. Considering that the PNPs in our study were unfamiliar products produced through an innovative service, and that consumers may initially approach such products with some degree of skepticism, the slightly positive sensory evaluations observed can be considered as a favorable outcome at this stage of development [37]. Beyond product acceptance, participants also provided generally favorable evaluations of implementation-related aspects of the PN service. Time investment, the amount of personal information that had to be shared, and the user-friendliness of the digital ordering application were evaluated as slightly positive. Previous research has shown that one of the challenges of PN services is that consumers may perceive the provisioning of personal information as time-consuming. In addition, user-friendliness has been identified as an important prerequisite for consumer engagement in personalized dietary advice tools and services [38,39]. In this study, participants did not perceive either the time investment or the user-friendliness of the PN service as barriers, which is an important factor for the implementation of this PN service. One implementation challenge identified in this study was the limited perceived variation in products throughout the intervention period, which received a slightly negative rating. This was primarily attributable to constraints of the prototype setup, as no alternative casings and fillings with comparable nutritional properties had been developed, rather than to inherent limitations of the 3D printing technology itself. Consequently, day-to-day product variation depended largely on the training program and the practical feasibility of the pilot study. Because the training program mainly contained heavy physical exercise, the nutrient-dense peanut butter filling was advised most often. Future implementation efforts should therefore prioritize an increase in product variety while maintaining nutritional tailoring.

A relatively high prevalence of GI complaints was reported after PNP consumption, affecting 36% of all participants. However, these findings should be interpreted with caution as total dietary intake of participants was not controlled during the study period, and the PNP constituted only a small proportion of participants’ overall food intake. In addition, it is likely that factors other than PNP consumption contributed to GI discomfort. The intervention period coincided with an intense training program, characterized by sleep deprivation and strenuous physical activities, both of which are known to affect GI function. GI complaints are commonly reported among soldiers during military missions and training exercises [21]. Finally, participants’ habitual low fiber intake at baseline may also have contributed to the occurrence of GI discomfort during the intervention period. Nevertheless, monitoring tolerance and GI complaints remains important during future implementation studies. Contrary to expectations, repeated consumption did not result in increased product liking over time. Although mean overall liking scores were descriptively higher at the end of the intervention than at baseline, no significant effect of time was observed. This finding differs from previous military research on 3D-printed foods [30]. However, unlike earlier studies in which participants repeatedly consumed the same product, the current intervention exposed participants to multiple personalized product variations. Consequently, familiarity with any single product remained limited, potentially attenuating a repeated-exposure effect.

Previous research on novel foods and food technologies suggests that repeated exposure can increase familiarity, which in turn may positively affect consumers’ attitudes [40,41]. In this study, participants reported positive attitudes at baseline; however, the mean scores for perceived added value and intentions to use PNPs across all 3 behavioral stages (e.g., precontemplation, contemplation, and action) decreased significantly over time toward a more neutral or slightly positive score at the end of the study. A similar pattern was observed for perceived potential health benefits of both the products and the service. Ratings decreased from positive at baseline to more neutral at the end of the study for immune health, recovery, endurance, and muscle power. Only perceived digestive health benefits remained slightly positive throughout the intervention period. The observed may be explained by initially high expectations regarding personal health benefits, even though this was out of the scope of this study.

The decline in attitudes and perceived health benefits suggests that the current prototype was not able to sustain the initially favorable user evaluation over time. Therefore, further optimization of both the products and service components appears necessary to support long-term acceptance and continued engagement with the PN service (Potential strategies for optimization of the PN service section).

An encouraging finding for future implementation was the absence of substantial concerns regarding sharing personal information for the creation of personalized food products. Although privacy risks are frequently cited as a barrier to the adoption of PN, and previous studies have shown that many consumers perceive data sharing as a risk that outweighs potential benefits [42,43], this was not observed in the present sample. Although this finding may partly reflect the military context and should be interpreted with caution, it suggests that data-sharing requirements are unlikely to constitute a major implementation barrier in similar settings.

Finally, interpretation of the findings should consider the challenging operational context in which the intervention was conducted. Participants underwent an intensive training program, characterized by prolonged physical exertion, outdoor exercise, and severe sleep restriction. These circumstances may have influenced both well-being and product evaluations. However, simultaneously, the feasibility of deploying and evaluating a PN service under realistic military conditions has been demonstrated.

### Strengths and limitations

A major strength of this study was the evaluation of a PN service under real-world conditions in a military setting. Rather than assessing products in a controlled laboratory environment, participants interacted with the service and consumed the PNPs during their regular training activities. This approach provided insight into the feasibility of implementing a PN service in practice and generated user experiences that are likely to reflect real-world use and acceptance.

Another strength lies in the evaluation of the whole PN service. All components were tested and assessed, ranging from the personalized advice itself, ordering a PNP including a flavor selection, to the production of the PNPs and the subsequent sensory experience. Finally, the study design incorporated repeated exposure to PNPs, which provided deeper insights into longer-term user perceptions. Unlike single-exposure studies, repeated exposure allowed assessments of changes in product appreciation and attitudes toward the service over time.

Several limitations should also be acknowledged. First, this study evaluated an early prototype of the PN service, and further optimization of both the products and service components is warranted. Product variety was limited, partly because food materials were developed to serve multiple target populations rather than being optimized specifically for military personnel [17]. In addition, the FFMP was able to produce a limited number of 25 products per day, and therefore, the service could only be evaluated in a relatively small sample. Furthermore, the prototype nature of the PNPs, their use as a single in-between meal, and the military study setting (in a male-only peloton) limit the generalizability of the findings. Nevertheless, the implementation insights generated by this study could still be relevant for the development and evaluation of PN services in other real-world contexts.

Second, no control group was included, as the primary objective of this proof-of-concept study was to evaluate implementation feasibility and consumer acceptance. Consequently, the study does not allow us to draw conclusions on the comparative effectiveness or added value of the PN service relative to conventional nutrition support, nor can causal conclusions be drawn regarding observed changes in attitudes, intentions, or well-being. However, the pretest–posttest design does allow assessment of our implementation-related outcomes, including successful deployment of the service, user experiences, product liking, and within changes over time.

Third, user experiences were assessed using subjective questionnaires, which are inherently subject to limitations, such as response bias and socially desirable answering behavior [44,45]. Furthermore, the personalized nature of the intervention created challenges in the statistical analyses. PN is fundamentally an N-of-1 approach, whereas conventional statistical methods rely on mean differences within or between groups. Because product allocation depended on individual nutrition requirements, participants received different product combinations and consumed them with varying frequencies. This unbalanced design limited the ability to examine changes in liking for individual product types over time. Another limitation concerns the incomplete disclosure of 3D printing technology. Although participants were not explicitly informed about the use of 3D printing technology in the production process (incomplete disclosure), all participants reported in the endline questionnaire that they were aware that the products had been 3D printed. This awareness may have influenced objective sensory evaluations. Previous research has shown that novel food technology neophobia can affect willingness to try novel foods and influence product evaluations [29,30]. However, military personnel have been reported to perceive 3D-printed foods relatively positively and to be less susceptible to such neophobia [30].

### Potential strategies for optimization of the PN service

Given the fact that positive user attitudes toward the PN service and potential health benefits declined toward more neutral attitudes by the end of the study, several optimization steps need to be taken before implementation of PNP at a larger scale can be considered. First, increasing the visibility of the personalization process may strengthen users’ perception of relevance and added value. During the study, participants received information on the health benefit associated with their PNP and the reason why this benefit was assigned to them. However, this information was only displayed briefly when participants ordered a new PNP on a tablet for the following day and was not available when the product was consumed. As a result, the link between participants’ personal characteristics, daily input data, and the nutritional rationale underlying the PNP may not have always been sufficiently visible. Future versions of the service could therefore provide personalized feedback at multiple timepoints, including at the time of product consumption. For example, product packaging, labels, quick response codes, or a mobile application could be used to remind users why a specific product was recommended, which personal data contributed to the recommendation, and which health benefit the product is intended to support.

Second, increasing product variety and sensory quality is likely to improve acceptance. Product variation received the lowest rating of all service aspects (slightly negative) and was constrained by the limited number of formulations available during this prototype phase. In addition, participants provided feedback related to the sensory characteristics of the PNPs, particularly regarding filling and texture. Future development should therefore not only focus on expanding the portfolio of product combinations but also on further optimization of the product sensory quality. A first step could be to build upon the product combinations that received the most favorable evaluations in the current study and use these as a basis for further product development. The flexibility of 3D food printing technology offers opportunities to develop a much broader range of fillings, flavors, textures, shapes, and product formats than was available in the current study. Finally, users could be provided with more choices in the personalization process. Rather than only selecting a topping flavor, participants could be offered a choice between multiple nutritionally equivalent filling types, flavor combinations, or product formats. Together, these improvements may contribute to higher perceived value, stronger engagement, and more sustained acceptance of the PN service.

### Recommendations for future research

The present study focused on implementation feasibility and consumer acceptance rather than efficacy. Controlled intervention trials are therefore required to assess the added value of PN for health improvement, particularly as consumers appear more receptive to 3D-printed foods and other food innovations when clear personal health benefits are evident [46]. Future studies should preferably include an appropriate comparator condition, such as commercially available healthy snacks, combined with generic dietary advice. In a military context, it is important to study the effects of PN on both physical and cognitive performance, as these domains are central to the rationale of our PN products, and yet remain insufficiently supported by randomized clinical trials. Generating such evidence is essential to determine whether personalized approaches can significantly improve operational readiness and resilience in demanding military environments.

In conclusion, this study supports the feasibility of implementing and evaluating a prototype PN service in a military setting. The study demonstrated that personalized dietary advice could be combined with the design, production, and distribution of tailored in-between meals in a demanding real-world environment. However, consumer acceptance of the PNPs was modestly positive, and initial positive attitudes toward the service and its perceived benefits declined over the study period. Therefore, further optimization of both the service and the products is needed to improve user evaluations, before implementation at a larger scale can be considered. Future improvements should focus on increasing the visibility of personalization and health feedback throughout the user journey, optimizing the sensory quality of the products, as well as expanding product variety and user choice.

## Authors contributions

The authors’ responsibilities were as follows – SvdH, MU, FH, SW, MN: contributed to conceptualization; SvdH, MU: contributed to data curation and investigation; SvdH: contributed to formal analysis; SvdH, MU, FH: contributed to writing—original draft; IWKK, MN, SW: contributed to writing—review and editing; and all authors: contributed to methodology.

## Data availability

The datasets used and analyzed during the current study are available from the corresponding author on reasonable request.

## Ethics approval and consent to participate

All participants provided signed informed consent for inclusion in the study. Ethical clearance for the study was obtained from the Wageningen University Research (WUR) Research Ethics Committee (REC) under approval number 2024-041.

## Declaration of generative AI and AI-assisted technologies in the writing process

During the preparation of this work, the authors used Microsoft 365 Copilot within the Wageningen University & Research Microsoft 365 environment exclusively for language editing and sentence refinement. After using this tool, the authors reviewed and edited the content as needed and take full responsibility for the content of the publication.

## Funding

The research carried out within this public–private partnership “IMAGINE” is funded by the Ministry of Economic Affairs, through Top Sector Life Sciences & Health, grant numbers 21074 and 100340397 and through Top Sector High Tech Systems and Materials, grant number 100340397. Partners in this public–private partnership are: Wageningen University & Research (WUR), Netherlands Organization for Applied Scientific Research (TNO), Dutch Ministry of Defence, Hospital Gelderse Vallei, GEA, Solipharma, Tate & Lyle, Nissin, and General Mills.

## Conflict of interest

The authors report no conflicts of interest.
